# Age-Related Structural and Functional Changes in the Mouse Lung

**DOI:** 10.3389/fphys.2019.01466

**Published:** 2019-12-04

**Authors:** Henri Schulte, Christian Mühlfeld, Christina Brandenberger

**Affiliations:** ^1^Institute of Functional and Applied Anatomy, Hannover Medical School, Hanover, Germany; ^2^Cluster of Excellence REBIRTH (From Regenerative Biology to Reconstructive Therapy), Hanover, Germany; ^3^Biomedical Research in Endstage and Obstructive Lung Disease Hannover (BREATH), Member of the German Center for Lung Research (DZL), Hanover, Germany

**Keywords:** pulmonary aging, micromechanics, stereology, late alveolarization, alveolar epithelial type II cells

## Abstract

Lung function declines with advancing age. To improve our understanding of the structure-function relationships leading to this decline, we investigated structural alterations in the lung and their impact on micromechanics and lung function in the aging mouse. Lung function analysis was performed in 3, 6, 12, 18, and 24 months old C57BL/6 mice (*n* = 7–8/age), followed by lung fixation and stereological sample preparation. Lung parenchymal volume, total, ductal and alveolar airspace volume, alveolar volume and number, septal volume, septal surface area and thickness were quantified by stereology as well as surfactant producing alveolar epithelial type II (ATII) cell volume and number. Parenchymal volume, total and ductal airspace volume increased in old (18 and 24 months) compared with middle-aged (6 and 12 months) and young (3 months) mice. While the alveolar number decreased from young (7.5 × 10^6^) to middle-aged (6 × 10^6^) and increased again in old (9 × 10^6^) mice, the mean alveolar volume and mean septal surface area per alveolus conversely first increased in middle-aged and then declined in old mice. The ATII cell number increased from middle-aged (8.8 × 10^6^) to old (11.8 × 10^6^) mice, along with the alveolar number, resulting in a constant ratio of ATII cells per alveolus in all age groups (1.4 ATII cells per alveolus). Lung compliance and inspiratory capacity increased, whereas tissue elastance and tissue resistance decreased with age, showing greatest changes between young and middle-aged mice. In conclusion, alveolar size declined significantly in old mice concomitant with a widening of alveolar ducts and late alveolarization. These changes may partly explain the functional alterations during aging. Interestingly, despite age-related lung remodeling, the number of ATII cells per alveolus showed a tightly controlled relation in all age groups.

## Introduction

Lung aging is accompanied by functional, micromechanical and structural alterations (Chan and Welsh, 1998; Sharma and Goodwin, 2006; Pinkerton et al., 2014). In the elderly (>65 years of age), lung diseases like chronic obstructive pulmonary disease (COPD; Ito and Barnes, 2009; Provinciali et al., 2011), fibrosis/IPF (Raghu et al., 2006; Collard, 2010; Fell et al., 2010; Thannickal, 2013), cancer (Howlader et al., 2019) or acute respiratory distress syndrome (ARDS; Ely et al., 2002; Rubenfeld et al., 2005) occur more frequently and with greater severity than in younger individuals. These pathologies are also associated with alterations in pulmonary structure and function. For better comprehension of pathological changes with age, it is therefore important to understand the changes of lung function, micromechanics, and structure during normal aging.

Lung function is described by parameters of physiological breathing (Becklake and Crapo, 1991; Pellegrino et al., 2005) that were observed to decline in the elderly (Janssens et al., 1999). For example, studies have shown an increased residual volume (RV) and functional reserve volume (FRV), as well as decreased values of forced expiratory volume in one second (FEV1) in elderly individuals (Kerstjens et al., 1997; Janssens et al., 1999; Turner et al., 2017). Moreover, static lung compliance was found to increase with age (Wahba, 1983; Levitzky, 1984). These changes lead to increased respiratory impairment in the elderly (Vaz Fragoso and Gill, 2012). The mechanical properties of the lung tissue that influence lung function are described by different parameters, such as lung stiffness and elastance (Suki et al., 2011; Suki, 2014; Suki and Bartolák-Suki, 2014). Investigations on age-related changes in lung micromechanics have shown that the elastic recoil pressure decreases in the elderly (Janssens et al., 1999; Turner et al., 2017), possibly due to changes in tissue composition and remodeling (Mercer and Crapo, 1990; Toshima et al., 2004; Subramaniam et al., 2017). Changes in micromechanics are furthermore closely related to structural alterations in the human lung. Structural remodeling and airspace enlargement in aging human lungs was shown qualitatively (D’Errico et al., 1989) as well as quantitatively, by estimation of mean linear intercept [L_m_] (Verbeken et al., 1992a). In an MRI-based study with hyperpolarized ^3^He gas, Quirk et al. (2016) further confirmed an increase of L_m_ as well as decreasing alveolar depth and larger alveolar ductal radius in aging human lungs. These findings show that lung function, micromechanics and structure are closely linked and age-dependent. However, most studies on lung function and micromechanics in humans are cross-sectional and non-invasive and cannot provide the informational quality as research conducted under controlled laboratory conditions. Furthermore, histological samples of healthy human lung tissue are difficult to obtain and often not suited for quantitative structural analysis by means of stereology (Hsia et al., 2010) due to inadequate sample preparation. Thus, studies investigating lung aging are frequently performed under controlled laboratory conditions in animal models to correlate functional and structural parameters in the lung (Zosky, 2015).

In mice, the assessment of lung function and micromechanics is often done by forced ventilation techniques and measurements of pressure-volume changes (Vanoirbeek et al., 2010; De Vleeschauwer et al., 2011; Robichaud et al., 2017). Previous studies on lung function over different time courses of maturation and aging provided evidence of an increased inspiratory capacity (IC) (including tidal volume and inspiratory reserve volume) or static lung compliance with age (Huang et al., 2007; Veldhuizen et al., 2019). Major changes in lung function were further found in early adulthood and to a lesser extent later in life (Zosky, 2015; Elliott et al., 2016). Micromechanically, elastic recoil pressure and airway resistance were shown to decrease with age in mice, whereas lung tissue stiffness tended to increase (Huang et al., 2007; Elliott et al., 2016; Veldhuizen et al., 2019).

Structural changes of the mouse lung with maturation and age included stereological quantification of parameters that are relevant for lung function such as lung volume, alveolar volume and number as well as septal surface area and thickness. However, results on structural changes with age are controversial. For example, Pozarska et al. (2017) found the highest alveolar numbers in 9 months old (mo) mice and biggest alveoli in 22 mo mice in an experimental study covering an age range of 2, 9, and 22 mo mice, whereas another study found no difference in alveolar number in 6 and 24 mo mice (Glassberg et al., 2014). Moreover, little is known about changes in parenchymal airspaces, alveolar septal surface area and volume in the course of aging. These parameters contribute to intact lung function and mechanics and changes may affect the integrity of the lung (Subramaniam et al., 2017; Knudsen and Ochs, 2018). At cellular level, alveolar epithelial type II (ATII) cells play an important role for intact lung function as they produce alveolar surface tension reducing surfactant to prevent alveolar collapsing during breathing (Bachofen and Schürch, 2001). However, no data is available on ATII cell number and size in pulmonary aging. Additionally, although structural investigation by design-based stereology is considered as the gold-standard for morphometric lung research (Hsia et al., 2010), little information is available linking lung functional and micromechanical measurements with profound stereological analyses of the aging mouse lung.

Thus, the aim of this study was to contribute to a comprehensive understanding of age-related structural changes in the mouse lung and linking the findings to functional and micromechanical parameters. As most investigations on lung aging are based on only two age groups (Glassberg et al., 2014; Kling et al., 2017; Veldhuizen et al., 2019), the understanding of gradual changes in lung aging is still limited. The present study investigated age-related changes in lung function, micromechanics and structure in male C57/BL6JRj mice aged 3, 6, 12, 18, or 24 months to provide a more comprehensive picture of the developmental process from young adulthood to later stages of life.

## Materials and Methods

### Animal Model

Male C57BL/6JRj mice were ordered from Janvier Labs (France) at an age of 3, 6, 12, 18, or 24 months, including 7–8 animals per age group. Before experimental start the animals were allowed to acclimate for 1 week at the local housing facility (Zentrales Tierlaboratorium, Hannover Medical School) with food and water *ad libitum*. The experiments included lung function measurements and quantitative light microscopic analysis (stereology) of the lung. Animal procedures were authorized by the responsible authorities at LAVES (Niedersächsisches Landesamt für Verbraucherschutz und Lebensmittelsicherheit) and are in accordance with the German law for animal protection (TierSchG; BGBl. I, p. 1206 with corr. on p. 1313) and with the European directive, 2010/63/EU.

### Lung Function and Micromechanics Measurements

Before lung function testing, mice were injected with 80 mg/kg body weight ketamine (Anesketin 100 mg/ml, Eurovet Animal Health B.V.) and 5 mg/kg body weight xylazine (Rompun 2%, Bayer Vital GmbH) intraperitoneally. Under deep anesthesia, tracheotomy was performed and the animals were ventilated by a mechanical ventilation system (flexiVent^®^ FX Module 1 for mice, SCIREQ^®^ Scientific Respiratory Equipment Inc.) at 100 breaths/min and a tidal volume of 10 ml/kg body weight. To suppress spontaneous breathing during lung function measurements, the mice received 0.8 mg/kg body weight pancuronium bromide (Pancuronium-Actavis 2 mg/ml, Actavis GmbH) intraperitoneally. Lung function measurements were taken at a constant positive end-expiratory pressure (PEEP) of 3 cmH_2_O after two recruitment maneuvers. In a different set of animals aged 3, 6, or 18 months (*n* = 7/age), derecruitability maneuvers were performed with increasing PEEPs of 1, 3, 6, and 10 cmH_2_O. The aim of this experiment was to test for age differences in micromechanical behavior under non-physiological conditions. Static compliance (Cst) was computed by the Salazar-Knowles equation (Salazar and Knowles, 1964). Micromechanical parameters such as tissue resistance (G) and tissue elastance (H) were assessed by forced oscillations following an established protocol (Lutz et al., 2015; Lopez-Rodriguez et al., 2016). Tissue hysteresivity (η) was computed as the ratio of G/H and hysteresis was calculated as the area between the inflation and deflation limbs of the pressure-volume curve.

### Lung Fixation and Sample Preparation

After lung function measurements mice were killed and the lungs were fixed by tracheal instillation at a pressure of 20 cmH_2_O with aldehyde fixative, containing 1.5% paraformaldehyde (Sigma-Aldrich) and 1.5% glutaraldehyde (Sigma-Aldrich) in 0.15 M HEPES buffer (Merck Millipore). Afterward the heart–lungs-package was excised from the body and stored in the fixative for at least 24 h. Lung volume [V(lung)] was estimated using the Archimedes principle (Scherle, 1970; Schneider and Ochs, 2013). After lung volume measurements, lungs were consecutively sectioned into slices. Every other slice was embedded in glycol methacrylate (Technovit^®^ 7100, Kulzer GmbH), while the remaining samples were subsampled and embedded in epoxy resin (Glycid ether 100 for electron microscopy, SERVA Electrophoresis GmbH). Both embedding protocols were conducted as previously described in detail (Schneider and Ochs, 2014).

From the glycol methacrylate embedded samples 1.5 μm thick sections were cut and stained with toluidine blue (Toluidinblau O, C.I. 52040, Carl Roth GmbH) and eosin-orcein (Eosin G, C.I. 45380, Sigma-Aldrich and Orcein, C.I. Natural Red 28, Sigma-Aldrich), respectively. From the epoxy resin embedded samples sections with a thickness of 1 μm were cut and stained with toluidine blue.

### Design-Based Stereology

Design-based stereology is the gold standard for quantitative morphometric analyses of the lung due to its accuracy, efficiency, and precision (Hsia et al., 2010) and was applied throughout the entire study. The investigator was blinded to the group identity of the samples during the analysis. The tissue slides were digitalized with a light-microscopic slide scanner (AxioScan.Z1, Carl Zeiss Microscopy GmbH). Systematic uniform random sampling (SURS) (Gundersen and Jensen, 1987; Gundersen et al., 1999; Nyengaard and Gundersen, 2006; Hsia et al., 2010) for stereological analysis was performed with the newCAST^TM^ software (version 5.3.1.1640, Visiopharm^®^). The following parameters were estimated by stereology: volumes of lung parenchyma, non-parenchyma, septum, parenchymal airspace, alveolar airspace, and ductal airspace. Moreover, septal surface area, number of alveoli, and number and volume of ATII cells were quantified as previously described in detail (Brandenberger et al., 2015).

The estimation of the parenchymal lung volume [V(par,lung)] and non-parenchymal lung volume [V(non-par,lung)] was done by point counting on toluidine blue stained, glycol methacrylate embedded sections. SURS was performed with the Visiopharm^®^ software at a magnification of 5× and a sampling fraction of 15–25%. Sampling fractions were dependent on the size of the embedded lung sections and kept constant within one animal. A stereological test grid with 36 sampling points was used (Supplementary Figure 1A). Calculation was done by multiplication of the volume density [V_V_(par/lung)] with the total lung volume [V(lung)] as shown in Eq. 1:

(1) V⁢(par,lung)=∑P⁢(par)/∑P⁢(tot)⋅V⁢(lung)

While ΣP(tot) represents all points falling on lung tissue, ΣP(par) represents the points on parenchymal tissue. Corresponding calculations were performed for non-parenchymal volume estimation.

Total parenchymal airspace volume [V(airtot,par)], septal volume [V(sept,par)], total septal surface area [S(sept,par)] and mean septal thickness [$\bar{\text{τ}}$(sept)] were quantified on toluidine blue stained, glycol methacrylate embedded sections, scanned with an objective lens magnification of 20×. SURS was performed at sampling fractions of 1.25 to 3.5% per animal using a 20× magnification. The stereological test system contained 12 test lines with 24 test points and a length per point [l/p] of 9.4 μm (Supplementary Figure 1B). Point counting was applied to estimate septal volume density [V_V_(sept/par)] and total parenchymal airspace volume density [V_V_(airtot/par)]. Septal volume [V(sept,par)] and total airspace volume [V(airtot,par)] were achieved by multiplying the respective volume densities with V(par,lung).

The septal surface density [S_V_(sept/par)] was estimated by counting intersections (I) of test lines with the septal surface. S(sept,par) was calculated by multiplying S_V_(sept/par) with the reference volume as described previously (Weibel, 1979; Cruz-Orive and Weibel, 1990; Mayhew, 1991; Howard and Reed, 1998; Schneider and Ochs, 2013) and shown in Eq. 2:

S⁢(sept,par)=2⋅∑I⁢(sept)/(l/p⋅∑P⁢(tot))

(2) ⋅V(par,lung)

Last, the septal thickness [$\bar{\text{τ}}$(sept)] was calculated as shown in Eq. 3:

(3) $$\bar{\text{τ}}\,\left(\text{sept}\right)=2\cdot V_{\text{V}}\,\left(\mathrm{sept}/\mathrm{par}\right)/S_{\text{V}}\,\left(\mathrm{sept}/\mathrm{par}\right)$$

To distinguish alveolar airspace [V(airalv,par)] and ductal airspace [V(airduct,par)], a further investigation was done on toluidine blue stained, glycol methacrylate embedded sections scanned with an objective lens magnification of 20×. SURS was performed at a sampling fraction of 2.5–6% per animal and a magnification of 10×. The stereological test grid contained 36 points (Supplementary Figure 1C). Calculations of ductal volume density [V_V_(airduct,par)] and volume V(airduct,par) were done analogously to parenchymal volume estimations, as shown in Eq. 1. The alveolar airspace volume [V(airalv,par)] was calculated by subtraction of V(airduct,par) from V(airtot,par).

For the unbiased estimation of alveolar number [N(alv,par)] and ATII cell number [N(ATII,par)], the physical disector was used (Sterio, 1983; Ochs, 2006). This method allows the estimation of numbers in a test volume by creating a 3D test system from two planar and parallel cut sections. An unbiased counting frame was used for number estimations (Gundersen, 1977).

Alveoli are assumed to have one entrance to alveolar ducts which is enclosed by a ring of elastin fibers (Mercer and Crapo, 1990; Toshima et al., 2004; Meyerholz et al., 2017). The number of alveoli can be estimated by the Euler number, which describes the connectivity of the alveolar network (Hyde et al., 2004; Ochs et al., 2004). In principle, three topological events are possible to describe a network: bridges, islands, and holes. In the case of alveoli, the Euler number can be reduced to bridges as the other topological items occur very rarely (islands) or never (holes) (Gundersen et al., 1993). Thus, a counting event was the appearance of a closed alveolar elastin “bridge” [B(alv)] on one section and two elastin tips (=open alveolar entrance ring) on the other section. For this analysis, glycol methacrylate embedded and eosin-orcein stained sections of two serial tissue sections with a disector height [h] of 3 μm were used (Ochs, 2006). This staining dyes elastin fibers in red-brown and thereby improves the visualization of bridges (Ochs, 2006). The slices were scanned with an objective lens magnification of 20× and further processed via SURS at a magnification of 20×. Sampling fractions of 2–4.75% per animal were used. The area of the counting frame [A] was 35,570 μm^2^ (Supplementary Figures 2A,B). Parenchymal reference space was measured using point counting [P(par)] on the lower left and upper right corners of the reference section. To increase efficiency, counting was applied in both directions of the image pair. Alveolar number was calculated by multiplying alveolar number density [N_V_(alv/par)] by the reference volume as shown in Eq. 4:

N⁢(alv,par)=∑B⁢(alv)/(∑P⁢(par)⋅A⋅h)

(4) ⋅V(par,lung)

The number-weighted mean alveolar volume [$\bar{\text{ν}}$_N_(alv)] was calculated as in Eq. 5:

(5) $$\,{\bar{\text{ν}}}_{\text{N}}\,\left(\mathrm{alv}\right)=V\,\left(\mathrm{airalv},\mathrm{par}\right)/N\,\left(\mathrm{alv},\mathrm{par}\right)$$

just like the number-weighted mean alveolar septal surface area [$\bar{S\,\left(\mathrm{sept}\right)/N\,\left(\mathrm{alv}\right)}$].

For the quantification of ATII cell numbers, nuclei of ATII cells [N(ATII)] were counted for the disector analysis as unique counting events per cell. Cells were counted, if their nucleus only appeared on one section of the disector image pair. Toluidine blue stained tissue sections of epoxy resin embedded samples were cut with a disector height [h] of 3 μm. Slices were scanned with an objective lens magnification of 40× and further processed via SURS at a magnification of 40×. Approximately 180 image pairs per animal (60 per slice) were generated and evaluated. The area of the counting frame [A] was 12,000 μm^2^ (Supplementary Figures 2C,D). The number of ATII cells [N(ATII,par)] was calculated by multiplication of the ATII cell number density [N_V_(ATII/par)] by the reference volume, as shown in Eq. 6:

N⁢(ATII,par)=∑N⁢(ATII)/(∑P⁢(par)⋅A⋅h)

(6) ⋅V(par,lung)

Simultaneously, the number-weighted mean volume of ATII cells [$\bar{\text{ν}}$_N_(ATII)] was estimated using the planar rotator (Vedel-Jensen and Gundersen, 1993). Only cells sampled within the disector pairs were subjected to the rotator, using the nucleus as reference point. From the individual estimations, the arithmetic mean was calculated to obtain the number-weighted mean volume of the cells.

### Statistical Analysis

All statistical analyses were performed using SigmaPlot^®^ software, version 13.0.0.83 (SYSTAT^®^ Software Inc.). Data were tested for normality by Shapiro–Wilk test and for equal variance by Brown-Forsythe test. If the data passed both tests, a one-way ANOVA was performed with a pairwise comparison by Bonferroni’s *t*-test. If the equal variance or normality test failed, data were ln transformed and a one-way ANOVA was performed afterward. For lung function measurements with increasing PEEPs, a repeated measures ANOVA with Bonferroni *t*-test was applied. No outlier tests were performed and no data were excluded from the study. Differences were regarded as significant if *p* < 0.05.

## Results

### Lung Function and Micromechanics Change Greatest in Early Mouse Adulthood

**FIGURE 1:**
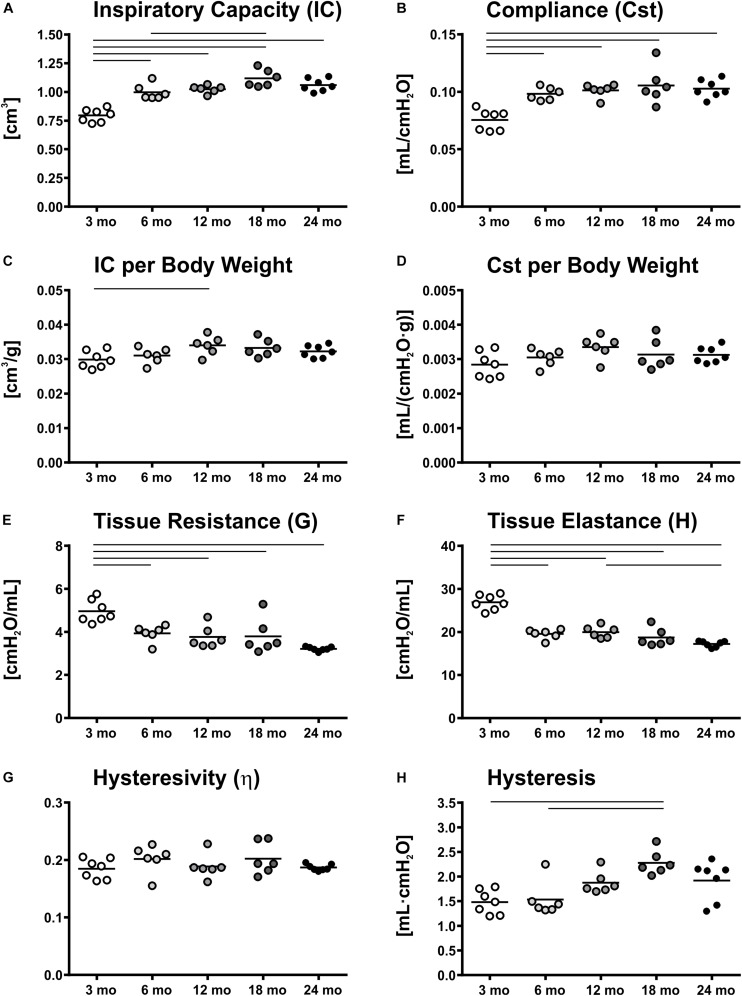
Lung function and micromechanics. Inspiratory capacity (IC) **(A)** and static compliance (Cst) **(B)** showed significant increases from young to middle-aged and old mice, respectively. However, IC and Cst corrected for body weight remained rather constant over lifetime **(C,D)**. Tissue resistance (G) **(E)** and tissue elastance (H) **(F)** changed most in early mouse adulthood. Hysteresivity (η) remained constant during aging **(G)**, whereas hysteresis increased from 3 and 6 mo mice to 18 mo mice **(H)**. Each point represents one animal; bars show means and lines indicate statistically significant differences between age groups (one-way ANOVA with Bonferroni *t*-test, *p* < 0.05).

The greatest changes in lung function such as inspiratory capacity (IC) and static compliance (Cst) were observed in the early period of adulthood, between young (3 mo) and middle-aged (6 and 12 mo) mice. Both, IC and Cst increased by 25% from 3 to 6 mo mice and reached their maximum values at 18 months (Figures 1A,B). However, body weight also increased with age, showing biggest changes between young and middle-aged mice (Table 1) and therefore, Cst and IC per body weight remained quite stable in the course of aging (Figures 1C,D). Only a small but significant increase in IC per body weight was found between 3 and 12 mo mice. Tissue resistance (G) and tissue elastance (H) decreased significantly from 3 to 6 months by 21 and 27%, respectively (Figures 1E,F), but no significant changes were found between middle-aged and old (18 and 24 mo) mice. Hysteresivity (η) showed no significant changes during aging (Figure 1G), whereas hysteresis increased significantly from 3 and 6 to 18 mo mice by approximately 50% (Figure 1H). All lung function measurement data are further displayed in Supplementary Table 1. Results of lung function measurements with increasing PEEPs of 1, 3, 6, and 10 cmH_2_O did not provide any further insights in age-related changes in micromechanics (Supplementary Figure 3).

**TABLE 1:** Data of body weight, lung volume, and stereological parameters regarding parenchymal volumes and alveolar septum.

**Parameter**	**3 mo**	**6 mo**	**12 mo**	**18 mo**	**24 mo**
m(body) [g]	26.7 (1.1)	32.4^∗^(1.5)	29.9^∗^(1.6)	33.8*(3.0)‡	32.7^∗^(2.0)
V(lung) [cm^3^]	0.97 (0.12)	1.01 (0.07)	1.02 (0.05)	1.35*(0.14)†‡	1.31*(0.07)†‡
V(lung)/m(body) [10^–2^ cm^3^/g]	3.65 (0.53)	3.13 (0.29)	3.42 (0.16)	4.04(0.54)†	4.01(0.38)†
V_V_(par/lung)	0.873 (0.025)	0.856 (0.023)	0.839 (0.034)	0.878 (0.027)	0.855 (0.025)
V_V_(non-par/lung)	0.120 (0.025)	0.139 (0.023)	0.156 (0.034)	0.116 (0.025)	0.140 (0.025)
V_V_(other/lung)	0.0074 (0.0037)	0.0062 (0.0016)	0.0067 (0.0036)	0.0063 (0.0029)	0.0063 (0.0030)
V_V_(airtot/par)	0.906 (0.013)	0.902 (0.012)	0.902 (0.011)	0.914 (0.008)	0.911 (0.012)
V_V_(airduct/par)	0.344 (0.029)	0.296^∗^(0.039)	0.291^∗^(0.016)	0.386(0.034)†‡	0.368(0.018)†‡
V_V_(sept/par)	0.094 (0.013)	0.098 (0.012)	0.098 (0.011)	0.086 (0.008)	0.089 (0.012)
V(par,lung) [mm^3^]	847 (101)	865 (71)	858 (69)	1190*(130)†‡	1119*(77)†‡
V(non-par,lung) [mm^3^]	117 (30)	140 (24)	158 (31)	157 (35)	182(31)*
V(airtot,par) [mm^3^]	767 (88)	781 (71)	774 (67)	1087*(121)†‡	1019*(73)†‡
V(airduct,par) [mm^3^]	292 (44)	257 (38)	249 (17)	460*(74)†‡	413*(44)†‡
V(airalv,par) [mm^3^]	475 (55)	525 (59)	525 (55)	627*(66)†‡	606^∗^(34)
V(sept,par) [mm^3^]	79.9 (16.7)	84.1 (6.5)	83.7 (8.1)	102.5^∗^(13.7)	99.7^∗^(13.7)
S_V_(sept/par) [mm^–1^]	63.8 (5.5)	65.0 (5.7)	66.6 (5.6)	59.7 (3.4)	56.1(6.4)†‡
S(sept,par) [10^3^ mm^2^]	54.1 (8.7)	56.1 (5.6)	57.0 (5.4)	71.1*(10.1)†‡	62.7 (7.8)
$\bar{\text{τ}}$(sept) [μm]	2.94 (0.22)	3.01 (0.29)	2.95 (0.33)	2.89 (0.19)	3.19 (0.31)

*Data are expressed as mean (SD). ^∗^*p* < 0.05 for 3 mo vs. 6, 12, 18, 24 mo; ^†^*p* < 0.05 for 6 mo vs. 12, 18, 24 mo; ^‡^*p* < 0.05 for 12 mo vs. 18, 24 mo.*

### Lung Parenchyma: Widening of Alveolar Ducts in Old Mice

**FIGURE 2:**
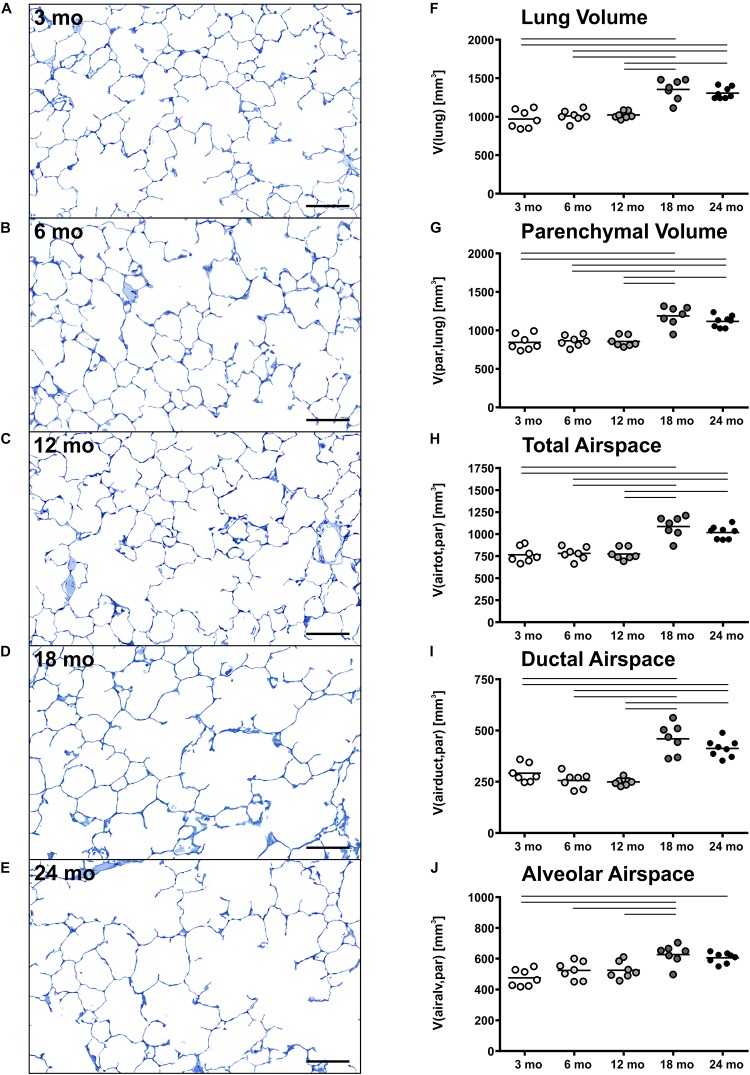
Lung parenchymal airspace volumes. **(A–E)** Representative light micrographs of each age group stained with toluidine blue show pulmonary histology. No inflammation or fibrosis was observed qualitatively at any time point. The light micrographs of 3 **(A)**, 6 **(B)**, and 12 **(C)** mo mice show a compact, regular lung parenchyma, whereas the light micrographs of 18 **(D)** and 24 **(E)** mo mice reveal visible widening of alveolar ducts causing a less compact appearance of the parenchyma. Scale bar = 100 μm. **(F–J)** Graphs show lung volume **(F)** and stereological results of parenchymal volume **(G)**, total parenchymal airspace volume **(H)**, ductal airspace volume **(I)**, and alveolar airspace volume **(J)**. Each point represents one animal; bars show means and lines indicate statistically significant differences between age groups (one-way ANOVA with Bonferroni *t*-test, *p* < 0.05).

Pulmonary histology of lung tissue revealed a normal lung parenchyma with absence of any pathological findings such as inflammation or fibrosis. Lung parenchyma from young and middle-aged mice appeared compact with many collateral alveoli surrounding narrow alveolar ducts (Figures 2A–C), whereas parenchyma of old mice appeared less densely packed due to wider alveolar ducts (Figures 2D,E). Stereological quantification revealed that lung volume, parenchymal volume as well as the volume of the parenchymal airspace increased approximately one third from middle-aged to old mice (Figures 2F–H), while the volume densities of these parameters remained almost constant (Table 1). The increase in the volume of the parenchymal airspace during aging was mainly driven by an increase in ductal airspace volume that occurred between 12 (249 mm^3^) and 18 mo (460 mm^3^) mice (Figure 2I), whereas the volume of the alveolar airspace just increased by 16% (Figure 2J). While the volume density of septal tissue remained constant in all age groups, the total septal volume increased from young to old mice (Table 1). Notably, no significant changes in total parenchymal, ductal and alveolar airspace volumes were measured between 18 and 24 mo mice.

### Increasing Number and Decreasing Size of Alveoli in Old Mice

**FIGURE 3:**
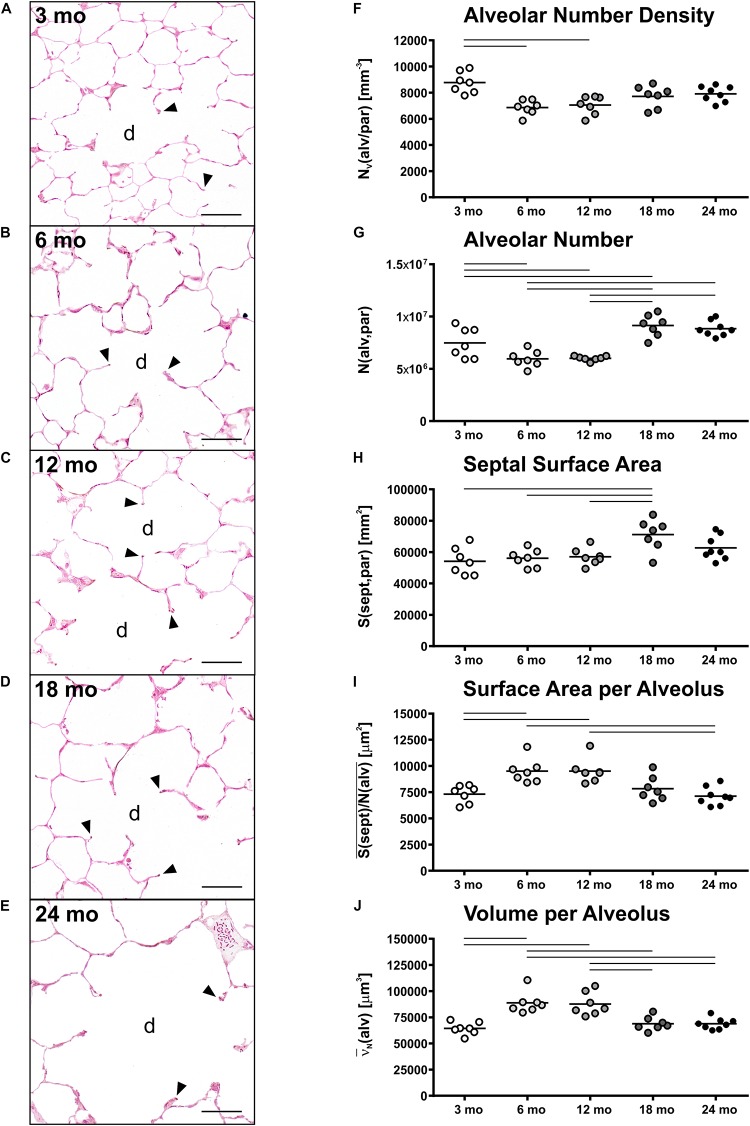
Alveolar changes with age. **(A–E)** Representative light micrographs of each age group show pulmonary histology of eosin-orcein stained lungs. Arrowheads indicate alveolar septal border tips as part of the alveolar entrance ring. Septal border tips are dyed brown by orcein, demonstrating elastin accumulation around the alveolar entrances. Alveolar ducts are indicated by “d.” At 3 months **(A)**, a high density of small alveoli and narrow alveolar ducts can be observed. The light micrographs of 6 **(B)** and 12 **(C)** mo mice show similar narrow alveolar ducts, but alveoli appear less dense. In 18 **(D)** and 24 **(E)** mo mice, the alveolar density seems to remain constant compared to middle-aged mice. The alveolar ducts, however, appear wider and alveolar septal borders seem to be shorter. Scale bar = 50 μm. **(F–J)** Graphs show stereological results of alveolar number density **(F)**, alveolar number **(G)**, total alveolar septal surface area **(H)**, mean septal surface area per alveolus **(I)**, and mean alveolar volume **(J)**. Each point represents one animal; bars show means and lines indicate statistically significant differences between age groups (one-way ANOVA with Bonferroni *t*-test, *p* < 0.05).

Histology of alveolar structures showed small and densely packed alveoli in young mice (Figure 3A), while the alveoli in middle-aged and old mice were more distended (Figures 3B–E). In eosin-orcein stained sections alveolar openings can be well detected due to brown staining of elastin tips at the end of septal borders. The septa oriented toward the alveolar duct thereby appeared shorter in old mice in comparison with middle-aged and young mice (Figures 3A–E). Stereologically, the number density of alveoli showed greatest and significant changes between young and middle-aged mice, decreasing with age (Figure 3F). The absolute number of alveoli on the other hand first decreased from young to middle-aged mice and then increased from middle-aged to old mice by approximately 50% (Figure 3G). The total septal surface area increased significantly from young to middle-aged and to old (18 mo) mice (Figure 3H). However, the septal surface density declined significantly between middle-aged and old (24 mo) mice (Table 1). Accordingly, the mean septal surface area per alveolus was approximately one third bigger in middle-aged compared to young or old (24 mo) mice (Figure 3I). Similarly, the number-weighted mean alveolar volumes were biggest in middle-aged mice (∼ 88,000 μm^3^) and significantly smaller in young (∼ 64,000 μm^3^) or old (∼ 69,000 μm^3^) mice (Figure 3J). The mean septal thickness remained relatively constant over the time course of aging at around 3 μm (Table 1).

### ATII Cell Number Density Remains Constant in Middle-Aged and Old Mice

**FIGURE 4:**
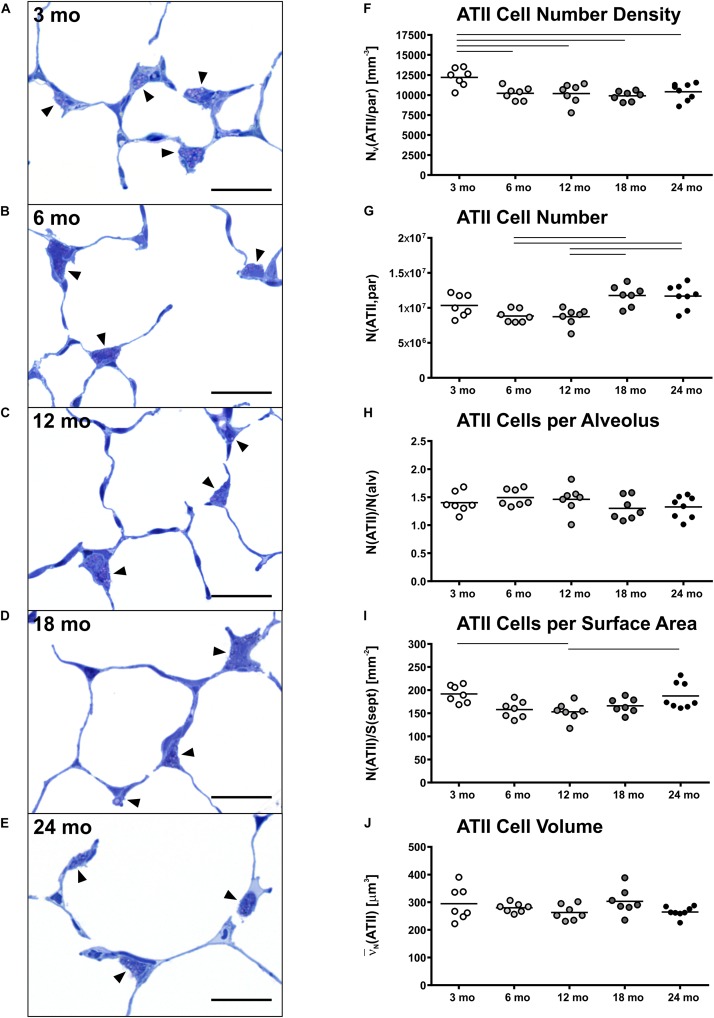
Alveolar epithelial type II (ATII) cell characteristics. **(A–E)** Representative light micrographs of each age group show pulmonary histology. Tissue sections were stained with toluidine blue. Arrowheads indicate ATII cells, typically located in alveolar “corners”. Intracellular lamellar bodies appear violet due to a metachromatic staining behavior. The ATII cells look quite homogeneous at any age in light microscopic images. However, the cell number density seems to be highest in 3 mo mice **(A)**. Scale bar = 20 μm. **(F–J)** Graphs show stereological results of ATII cell number density **(F)** and ATII cell number **(G)** as well as mean ATII cell number per alveolus **(H)**, mean ATII cell number per alveolar septal surface area **(I)**, and mean ATII cell volume **(J)**. Each point represents one animal; bars show means and lines indicate statistically significant differences between age groups (one-way ANOVA with Bonferroni *t*-test, *p* < 0.05).

Representative light micrographs of toluidine blue stained, epoxy resin embedded tissue sections with ATII cells are shown in Figures 4A–E. The ATII cells here are clearly distinguishable due to the metachromatic staining behavior of surfactant storing lamellar bodies. ATII cells were mostly localized in the “corners” of alveoli, near to branching septa and were part of the alveolar septum. Individual cell morphology at light microscopic level remained qualitatively uniform with age. In young mice (Figure 4A), the density of ATII cells was slightly higher compared to older mice (Figures 4B–E). This observation was also supported by stereological findings, as the number density of ATII cells was highest in 3 mo mice and significantly decreased with age, showing an approximately 20% lower density in old mice (Figure 4F). However, the absolute number of ATII cells increased from middle-aged (8.8 × 10^6^) to old mice (11.7 × 10^6^) (Figure 4G). Interestingly, the number of ATII cells per alveolus remained almost constant at a count of approximately 1.4 over the time course of aging (Figure 4H). The number of ATII cells per septal surface area declined from 3 to 12 months of age and increased again from 12 to 24 mo mice (Figure 4I). The mean volume of ATII cells, estimated by the planar rotator, stayed constant over lifetime at approximately 280 μm^3^ (Figure 4J).

## Discussion

The aim of this study was to investigate lung aging with respect to structure and function. The results show detailed structural and functional alterations in the mouse lungs over a broad time course from young adult to old mice. In summary, changes in lung function [inspiratory capacity (IC) and static compliance Cst], micromechanics (tissue resistance G and tissue elastance H) and alveolar number and size were greatest in early adulthood from 3 to 6 mo mice, whereas most other morphological parameters exhibited greatest alterations with progressing age in old mice. Particularly, the ductal airspace volume increased in old mice. Additionally, late alveolarization was observed as well as alveoli with shorter septa and a reduced alveolar depth. The number of ATII cells increased along with alveolar number in old mice.

### Lung Function and Micromechanics in Aging Mice

Greatest changes of lung function and micromechanics were observed in the early adulthood, between 3 and 6 months of age. This has also been shown by others for BALB/c (Ranga et al., 1979) and C57BL/6 mice (Elliott et al., 2016). While Huang et al. suggested a linear decline (Huang et al., 2007), our data supported a non-linear change in lung function and micromechanics in aging C57BL/6 mice, similarly as also shown by Elliott et al. (2016). They observed greatest alterations in early adulthood between 2 and 6 months of age, including increases in IC and Cst by 36% and decreases of G and H by 38% and 34%, respectively, but little changes were observed later in life (Elliott et al., 2016). These findings are consistent with our data showing similar increases for IC (+25%), Cst (+30%), G (−21%), and H (−27%) between 3 and 6 mo mice. Additionally, an increase in hysteresis was found from young and middle-aged (6 mo) to old (18 mo) mice (Figure 1H). Similarly, increases in hysteresis were found in elastase-induced emphysematous mouse lungs (Brewer et al., 2003; Vanoirbeek et al., 2010; Robichaud et al., 2017). Hysteresis is mainly affected by surfactant and the properties of extracellular matrix (ECM) (Suki et al., 2011). Little is known regarding surfactant function in later life, but in a previous study a decrease in septal elastin fibers and an increase in septal collagen fibers were measured between young (3 months) and old (18 months) mice (Kling et al., 2017), most likely contributing to the changes in pulmonary micromechanics with age.

In humans, Sicard et al. (2018) estimated the elastic modulus in different anatomical regions of the lungs via atomic force microscopy and found age-related increases in the stiffness of lung parenchyma and vessels. Furthermore, in humans many lung function parameters such as FEV1, forced vital capacity (FVC) and vital capacity (VC) show a decline with age (Ware et al., 1990; Verbeken et al., 1992b; Janssens et al., 1999; Sharma and Goodwin, 2006), while static lung compliance increases in the elderly (Wahba, 1983; Levitzky, 1984). The inter-species translation of lung function and micromechanics measurements from mice to humans or vice versa is difficult because of the different analytical procedures (Miller et al., 2005; Lopez-Rodriguez et al., 2016). Nevertheless, it seems as lung micromechanics are similarly altered in humans and mice with age.

### Structural Changes in the Lung Parenchyma With Progressing Age

Lung volumes increased with age, particularly in older mice (Figure 2F). This is in line with other studies in mice (Elliott et al., 2016; Pozarska et al., 2017). In humans, lung growth was reported to be mainly restricted to initial lung development and not occurring later in life (de Jong et al., 2006; Copley et al., 2009; Zach et al., 2012; Herring et al., 2014), except after hemi-pneumonectomy (Butler et al., 2012). This finding, however, suggests that lung growth can occur throughout lifetime.

Accompanied by an increase in lung volume in old mice, an enlargement of alveolar ducts was observed (Figure 2I). This has been described before in aging mammalian lungs. Beside qualitative observations (D’Errico et al., 1989; Kerr et al., 1990; Yamamoto et al., 2003; Glassberg et al., 2014), mean linear intercept [L_m_] is a frequently used parameter to estimate the mean chord length of the complete acinar airspace complex (Verbeken et al., 1992a; Huang et al., 2007; Elliott et al., 2016; Quirk et al., 2016). L_m_ is inversely related to S/V as 4/L_m_ and expresses the mean free length between two intersections of alveolar septal surface with an unbiased test line system of straight lines (Knudsen et al., 2010). Although this parameter is easy to assess, it cannot reflect different compartments of air-filled spaces. Furthermore, L_m_ is susceptible to bias as it does not consider shape and critically depends on the execution of lung inflation, lung volume measurement, and (unbiased) sampling procedures in histological studies, as reviewed in Weibel et al. (2007). Therefore, L_m_ cannot be stated as a sufficient parameter for parenchymal airspace alterations (Weibel et al., 2007; Mühlfeld and Ochs, 2013). Only with estimation of alveolar number and by differentiating between alveolar and ductal airspace, the correct and unbiased conclusions can be drawn on changes in alveolar size and ductal airspace (Mühlfeld and Ochs, 2013). Nevertheless, comparing our results of septal surface density S_V_(sept/par) or, practically, S/V in older mice with studies in aging C57BL/6 mice estimating L_m_ (Huang et al., 2007; Elliott et al., 2016), their findings of increasing L_m_ with age is in line with our data of decreasing S_V_(sept/par). Considering a concomitant increase in alveolar number density N_V_(alv/par) with age, this provides evidence of declining alveolar size, which is coherent with enlargement of the alveolar ducts.

### Age-Related Changes in Alveolarization

Alveolarization is mainly a postnatal process and occurs as early “bulk” and a late “continued” alveolarization (Schittny, 2017). Late alveolarization is discussed as the formation of new alveoli after the maturation of the alveolar septum which outlasts later periods of lung development (Burri, 2006; Schittny et al., 2008; Tschanz et al., 2014). Alveolarization has been shown to continue until young adulthood in humans (Narayanan et al., 2012; Herring et al., 2014), rats (Tschanz et al., 2014) and mice (Mund et al., 2008; Pozarska et al., 2017), however, little is known about late alveolarization in the elderly or in aged rodents.

The investigations showed that the number of alveoli increased from middle-aged to old mice, suggesting a late alveolarization in the old animals. Similarly, a study comparing 3 and 20 mo mice via microCT showed an increased number of alveoli per acinus in old mice (Vasilescu et al., 2012), supporting the idea of late alveolarization as well. In this study, the mean septal surface area per alveolus increased by approximately 15% from 3 to 20 mo mice, which is coherent with our data displaying a slight, non-significant increase of 7% from 3 to 18 mo mice. However, the point in time with the most prominent late alveolarization in our study, occurring from middle-aged to old mice, was not covered in their study. Others also reported different findings. For example, Glassberg et al. (2014) found no age-related changes in alveolar number in 6 and 24 mo female C57BL/6 mice. Furthermore, a study from Pozarska et al. (2017), investigating postnatal lung alveolarization in juvenile and adult mice of both sexes with an age of 2, 9, or 22 months as adult groups, showed highest numbers of alveoli in 9 mo mice. Moreover, the study found alveoli to have greatest volumes in 22 mo mice, whereas our results showed a decrease in alveolar size in old mice. The opposing observations may occur due to strain and sex differences. Furthermore, the study presumably included the total parenchymal airspace volume V(airtot,par) comprising alveolar and ductal airspace volumes to estimate the number-weighted mean alveolar volume $\bar{\text{ν}}$_N_(alv) (Pozarska et al., 2017). In comparison, we only used the total alveolar airspace volume V(airalv,par) without ductal airspace to estimate $\bar{\text{ν}}$_N_(alv). This has a direct impact on the estimation of the mean alveolar volume. By excluding ductal airspace from the estimation of alveolar volume, changes in ductal airspace will have no impact on alveolar volume calculations (Table 2). Here, in our study we therefore found a decrease in number-weighted mean alveolar volume in old mice, while the total number of alveoli was still increasing with age.

**TABLE 2:** Data of stereological parameters regarding alveoli and ATII cells.

**Parameter**	**3 mo**	**6 mo**	**12 mo**	**18 mo**	**24 mo**
N_V_(alv/par) [10^3^ mm^–3^]	8.77 (0.81)	6.87^∗^(0.57)	7.04^∗^(0.71)	7.72 (0.84)	7.91 (0.59)
N(alv,par) [10^6^]	7.48 (1.44)	5.95^∗^(0.78)	6.00^∗^(0.23)	9.14*(1.05)†‡	8.84(0.72)†‡
N_V_(ATII/par) [10^3^ mm^–3^]	12.21 (1.17)	10.21^∗^(0.81)	10.18^∗^(1.28)	9.88^∗^(0.62)	10.41^∗^(1.07)
N(ATII,par) [10^6^]	10.34 (1.57)	8.82 (0.93)	8.73 (1.26)	11.76(1.50)†‡	11.68(1.73)†‡
${\bar{\text{υ}}}_{\text{N}}$(ATII) [μm^3^]	295 (61)	280 (17)	263 (28)	303 (48)	265 (19)
V(airalv,par)/N(alv,par) = ${\bar{\text{υ}}}_{\text{N}}$(alv) [10^3^ μm^3^]	64.5 (5.9)	88.8^∗^(10.2)	87.7^∗^(11.1)	69.0(6.5)†‡	68.9(5.3)†‡
V(airtot,par)/N(alv,par) [10^3^ μm^3^]	104.1 (10.2)	132.2^∗^(12.1)	129.3^∗^(14.7)	119.7 (13.8)	115.7 (8.9)
S(sept,par)/N(alv,par) = $\bar{S\,\left(\mathrm{sept}\right)/N\,\left(\mathrm{alv}\right)}$ [10^3^ μm^2^]	7.32 (0.8)	9.51^∗^(1.2)	9.52^∗^(1.2)	7.83 (1.2)	7.12(0.9)†‡
N(ATII,par)/S(sept,par) [mm^–2^]	192 (19)	158 (18)	153^∗^(20)	166 (16)	187(28)‡
N(ATII,par)/N(alv,par)	1.40 (0.18)	1.49 (0.15)	1.46 (0.24)	1.30 (0.21)	1.33 (0.20)

*Data are expressed as mean (SD). ^∗^*p* < 0.05 for 3 mo vs. 6, 12, 18, 24 mo; ^†^*p* < 0.05 for 6 mo vs. 12, 18, 24 mo; ^‡^*p* < 0.05 for 12 mo vs. 18, 24 mo.*

The findings of alveolar duct widening and smaller alveoli in old mice compared to middle-aged mice suggest a shortening of alveolar septal borders leading to a reduced alveolar depth, as described previously for aging human lungs (Quirk et al., 2016). The qualitative findings in pulmonary histology support this assumption (Figures 3D,E). As discussed by Quirk et al. (2016), the decreasing alveolar depth is concomitant with an increasing radius of alveolar ducts during aging in human lungs at 19–71 years of age. It is assumed that these observations correspond with decreasing elastic recoil pressure and increasing shear modulus of the lung tissue (Verbeken et al., 1992a; Subramaniam et al., 2017; Knudsen and Ochs, 2018). Furthermore, alterations in septal elastin and collagen fiber distribution are considered to contribute to these observations in old mice (Toshima et al., 2004; Subramaniam et al., 2017).

It has to be mentioned that age-related changes in mechanical behavior of the lungs, as observed in Cst, could also affect structural parameters such as alveolar size. This holds particularly true, if lungs are instillation fixed under a constant pressure. In our study, Cst increased from 3 mo mice to older mice (Figure 1C), therefore, it is possible that changes in Cst affected alveolar size differences between 3 mo and older mice. However, in middle-aged and old mice Cst remained quite constant (Figure 1C). Hence in these groups, we consider the results in alveolar size measurements comparable, showing an age-related decline in alveolar size in old mice.

### Alterations of ATII Cell Numbers With Progressing Age

ATII cells play an important role for the functionality of the lung, as they serve as progenitor cells for alveolar epithelial type I (ATI) cells, stabilize intra-alveolar surface tension by producing surfactant and have an immunological function (reviewed in Fehrenbach, 2001). While transcriptomics and proteomics were done in ATII cells of young and old mice (Angelidis et al., 2019) and age-related changes were assessed in ATII cells as reviewed in Brandenberger and Mühlfeld (2017), there is no research on the absolute ATII cell number at multiple time points of adulthood yet. Stereological ATII cell quantification is very time consuming due to the necessity of the disector, but is superior to other quantification methods like flow cytometry (Jansing et al., 2017; Dzhuraev et al., 2019) or planimetry (Jansing et al., 2017). Nevertheless, there is a lot of research quantifying ATII cells stereologically in human (Crapo et al., 1982; Stone et al., 1992), rat (Crapo et al., 1980; Stone et al., 1992) and mouse (Stone et al., 1992; Dzhuraev et al., 2019) lungs for one age group.

Our results showed slightly higher total and alveolar surface-related ATII cell numbers for young compared to middle-aged mice (Figures 4F,I). This is in line with investigations in early postnatal rat lung development, showing a decline in ATII cells in the first days of life (Kauffman et al., 1974) and appears biologically consistent, as ATII cells serve as progenitor cells for alveolar epithelial type I (ATI) cells (Evans et al., 1975). From middle-aged to old mice, the ATII cell number density remained quite constant (Figure 4G), whereas, unexpectedly, the total number of ATII cells increased significantly (Figures 4G,I). We hypothesized that ATII cell number would further decrease with age or remain at least constant due to progenitor cell senescence with aging (Alder et al., 2015; Chen et al., 2015). However, our results indicate that ATII cell number concomitantly increased with lung volume in old mice. The constant ratio of ATII cells per alveolus during aging supports this finding (Figure 4H). It appears possible that a constant ATII cell number per alveolus contributes to a maintaining surfactant secretion, preserving lung function in old mice (Hills, 1999; Fehrenbach, 2001). However, further research is needed to address this hypothesis.

### Developing and Aging of the Mouse Lung

C57BL/6 mice are commonly used for geriatric research (Miller and Nadon, 2000; Huang et al., 2007; Elliott et al., 2016; Kling et al., 2017; Angelidis et al., 2019). The median lifespan of male C57BL/6 mice is approximately 900 days or 29 months (Yuan et al., 2009). Flurkey et al. (2007) recommend using animals aged onto 85–90% of survivorship as oldest group for geriatric research, which would count for approximately 24 mo C57BL/6 mice. We summarized the 5 age groups of this study into 3 groups, because this classification reflected changes of almost all parameters and supported comprehension of the results: one young adult (3 mo), two middle-aged (6 and 12 mo), and two old (18 and 24 mo) mice groups. However, Flurkey et al. (2007) categorized 3–6 mo mice as mature adults and 10–14 mo mice as middle-aged. In recent aging studies with C57BL/6 (Kling et al., 2017; Brandenberger et al., 2018) and BALB/c (Gomez et al., 2007; Chen et al., 2014) mice, 3 and 18 mo groups are frequently used to represent young and old, respectively. Lung development occurs until young adulthood (Schittny, 2017). We found great changes in lung function, micromechanics, and structure between 3 and 6 months of age, suggesting lung development to continue beyond pubertal maturation. Supportively, Miller and Nadon (2000) discussed using mice of 4–6 months of age for young groups to avoid influences of post-pubertal maturation processes. Nevertheless, we considered it important to include also young adult mice of 3 months into our study to have the full range of adult life covered.

## Conclusion

In summary, this study provides comprehensive insights on structural alterations in the aging C57BL/6JRj mouse lung, linking it with changes in lung function and micromechanics. Age-related structural alterations mainly appeared between middle-aged and old mice and were characterized by lung growth accompanied by a widening of alveolar ducts. Moreover, late alveolarization occurred, concomitant with decreasing alveolar size, most likely due to reduced alveolar depth. Quite constant relations of ATII cells to alveolar number and alveolar surface area were observed. However, lung function and micromechanics altered most between young and middle-aged mice.

## Data Availability Statement

All datasets generated for this study are included in the article/Supplementary Material.

## Ethics Statement

The animal study was reviewed and approved by the Niedersächsisches Landesamt für Verbraucherschutz und Lebensmittelsicherheit (LAVES), Postfach 39 49, 26029 Oldenburg.

## Author Contributions

HS, CB, and CM designed the research, interpreted the results of experiments, edited and revised the manuscript, and approved final version of the manuscript. HS and CB performed the experiments, analyzed the data, and prepared the figures. HS drafted the manuscript.

## Conflict of Interest

The authors declare that the research was conducted in the absence of any commercial or financial relationships that could be construed as a potential conflict of interest.
