# Supplementary material for: Age-Related Structural and Functional Changes in the Mouse Lung
Source: Front Physiol. 2019 Dec 4;10:1466. doi: 10.3389/fphys.2019.01466 (PMC6904284; doi:10.3389/fphys.2019.01466)
Supplement: Supplementary file 1 [file Table_1.DOCX]

Supplementary Material

# Supplementary Tables

**Supplementary Table 1**. Data of lung function and micromechanics measurements.

| **parameter** | **3 mo** | **6 mo** | **12 mo** | **18 mo** | **24 mo** |
| --- | --- | --- | --- | --- | --- |
| Inspiratory Capacity (IC)  [cm^3^] | 0.80  (0.06) | 1.00*  (0.07) | 1.02*  (0.033) | 1.12*†  (0.08) | 1.06*  (0.06) |
| Compliance (Cst)  [10^-2^ mL/cmH_2_O] | 7.55  (0.90) | 9.81*  (0.59) | 10.10*  (0.58) | 10.56*  (1.60) | 10.28*  (0.78) |
| IC/Body Weight  [10^-2^ cm^3^/g] | 2.99  (0.25) | 3.10  (0.23) | 3.40*  (0.28) | 3.33  (0.26) | 3.23  (0.18) |
| Cst/Body Weight  [10^-3^ mL/(cmH_2_O**·**g)] | 2.84  (0.38) | 3.05  (0.25) | 3.35  (0.33) | 3.13  (0.44) | 3.13  (0.23) |
| Tissue Resistance G  [cmH_2_O/mL] | 4.96  (0.53) | 3.93*  (0.39) | 3.76*  (0.52) | 3.79*  (0.81) | 3.22*  (0.09) |
| Tissue Elastance H  [cmH_2_O/mL] | 26.90  (1.74) | 19.55*  (1.14) | 20.00*  (1.37) | 18.72*  (2.08) | 17.23*‡  (0.69) |
| Hysteresivity η (G/H) | 0.185  (0.018) | 0.202  (0.025) | 0.189  (0.022) | 0.202  (0.028) | 0.187  (0.006) |
| Hysteresis  [mL**·**cmH_2_O] | 1.48  (0.25) | 1.53  (0.36) | 1.87  (0.22) | 2.28*†  (0.25) | 1.92  (0.40) |

Data are expressed as mean (SD). *p<0.05 for 3 mo vs. 6, 12, 18, 24 mo; †p<0.05 for 6 mo vs. 12, 18, 24 mo; ‡p<0.05 for 12 mo vs. 18, 24 mo

# Supplementary Figures

## Volume, surface, and thickness estimations

#
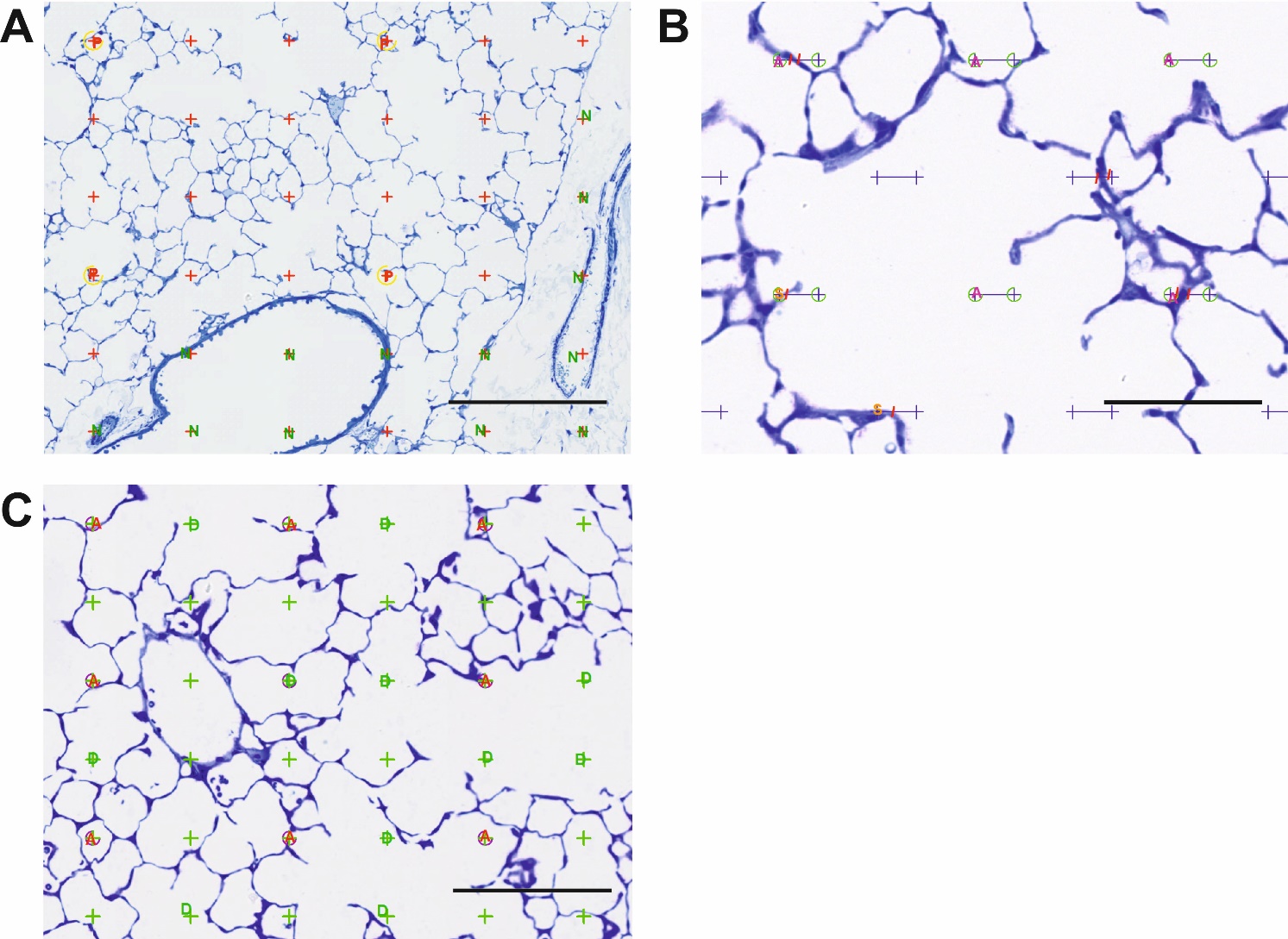


**Supplementary Figure 1.** Representative light micrographs for estimation of volume and surface densities using the Visiopharm® software. **(A)**: Parenchymal and non-parenchymal volume estimations were performed on tissue sections sampled at 5✕. The image was overlaid with a test grid containing 36 points and a subsampling factor of 9 was applied for parenchymal values. Scale bar = 300 μm. **(B)**: For the estimation of total parenchymal airspace volume density as well as septal volume and surface densities, 12 test lines of 9.4 μm with 24 test points were used. For alveolar airspace, a subsampling factor of 4 was applied. Sampling magnification was 20✕. Scale bar = 75 μm. **(C)**: Sampling for the differentiation of alveolar and ductal airspaces was done at a magnification of 10✕. A test grid containing 36 points and a subsampling factor of 4 for alveolar airspace were used. Scale bar = 150 μm.

## Number estimations

**
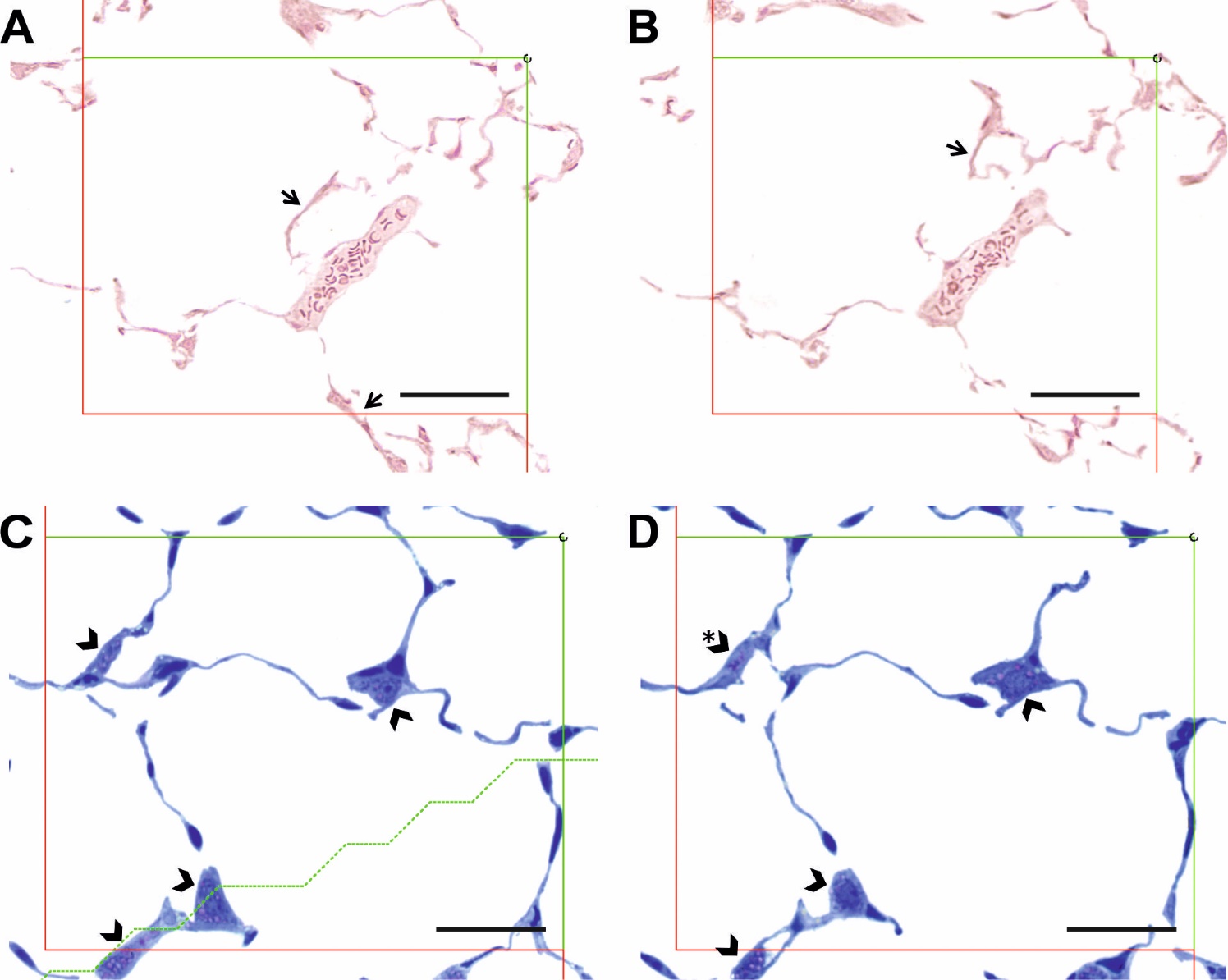
**

**Supplementary Figure 2.** Physical disector image pairs for estimating alveoli **(A,** **B)** or ATII cells **(C,** **D)**, respectively. The unbiased counting frames are defined by inclusion (green) and exclusion (red) lines. Arrows indicate newly formed ‘bridges’ **(A,** **B)**. Arrowheads indicate ATII cells and * indicates a counting event, as the ATII cells’ nucleus only appears on the right section **(C, D)**. Tissue sections of **(A)** and **(B)** were stained with eosin-orcein and scanned at an objective lens magnification of 20✕. Scale bar = 50 μm. Tissue sections of **(C)** and **(D)** were stained with toluidine blue and scanned at an objective lens magnification of 40✕. Scale bar = 25 μm.

## Lung function measurements with increasing PEEP


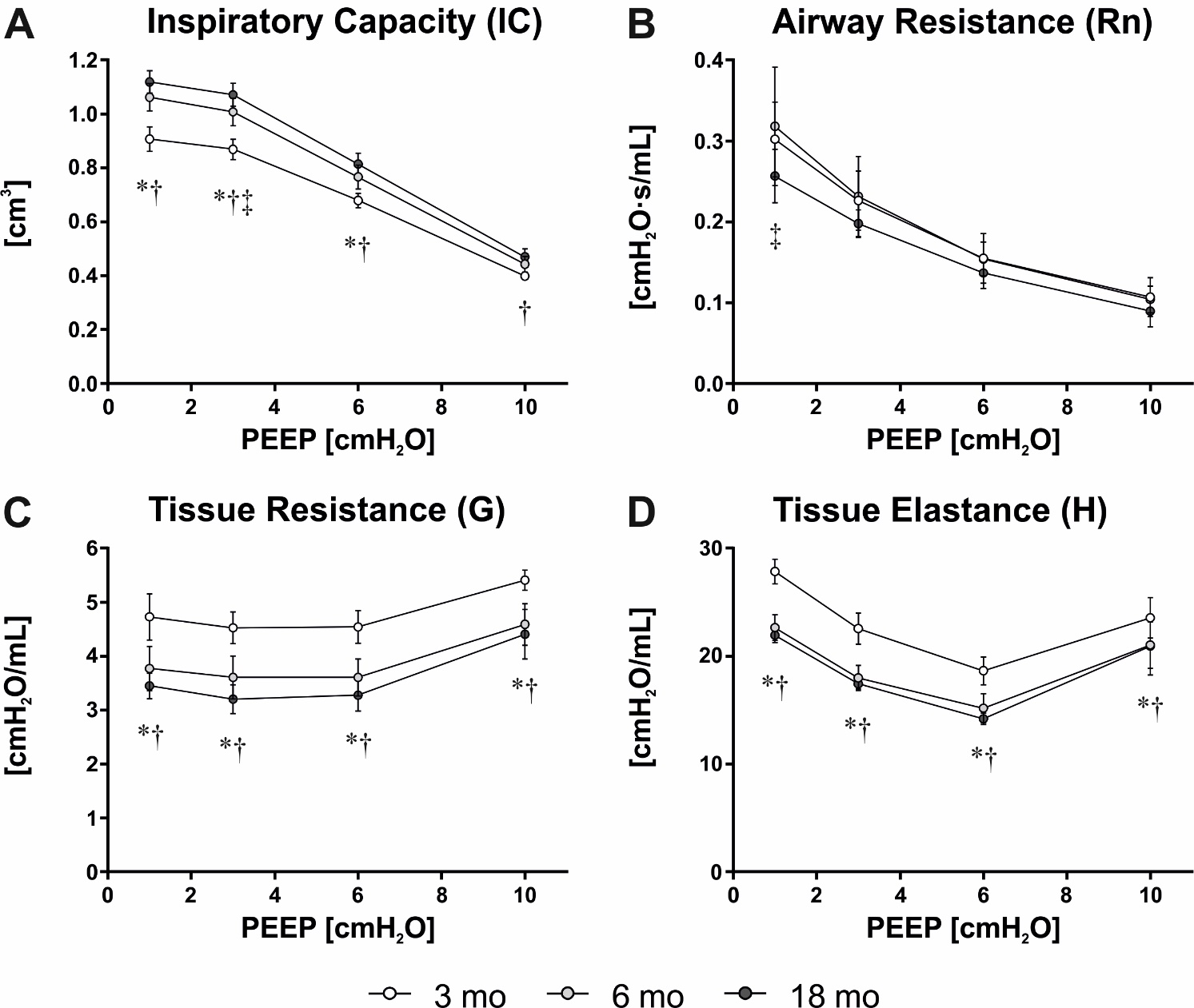


**Supplementary Figure 3.** Results of lung function measurements with increasing positive end-expiratory pressures (PEEPs) of 1, 3, 6, and 10 cmH_2_O in 3, 6, and 18 mo mice (n=7/age). Differences between age groups regarding inspiratory capacity (IC) **(A)** altered among different PEEPs. Airway resistance (Rn) **(B)** showed a significant decrease from 6 to 18 mo mice at PEEP 1 cmH_2_O, whereas at other PEEPs no significant age-related differences were detected. However, regarding tissue resistance (G) **(C)** and tissue elastance (H) **(D)**, significant differences were observed from 3 to 6 and 18 mo mice among all PEEPs. These results suggest a quite constant micromechanical behavior of same-aged mouse lungs among different PEEPs. Notably, at PEEP 3 cmH_2_O, age differences of IC, G, and H are consistent with lung function results of the main study population (fig. 1A, 1E, and 1F). Points show means of age groups per PEEP; error bars represent standard deviation (SD) in both directions; symbols indicate statistically significant differences between age groups per PEEP (repeated measures ANOVA with Bonferroni t-test, p<0.05) as follows: *p<0.05 for 3 mo vs. 6 mo; †p<0.05 for 3 mo vs. 18 mo; ‡p<0.05 for 6 mo vs. 18 mo.
